# Estradiol impairs the antiproliferative and proapoptotic effect of Zoledronic acid in hormone sensitive breast cancer cells in vitro

**DOI:** 10.1371/journal.pone.0185566

**Published:** 2017-09-25

**Authors:** Daphne Gschwantler-Kaulich, Sigrid Weingartshofer, Thomas W. Grunt, Mario Mairhofer, Yen Tan, Jutta Gamper, Christian F. Singer

**Affiliations:** 1 Department of Obstetrics and Gynecology, Comprehensive Cancer Center, Medical University of Vienna, Vienna, Austria; 2 Clinical Division of Oncology, Department of Medicine I, Comprehensive Cancer Center & Ludwig Boltzmann Cluster Oncology, Medical University of Vienna, Vienna, Austria; 3 Center for Advanced Bioanalysis GmbH (CBL), Linz, Austria; 4 QIMR Berghofer Medical Research Institute, Herston QLD, Australia; 5 Section for Medical Statistics, Center for Medical Statistics, Informatics, and Intelligent Systems, Medical University of Vienna, Vienna, Austria; University of South Alabama Mitchell Cancer Institute, UNITED STATES

## Abstract

**Background:**

Zoledronic acid (ZA) has antiresorptive effects and protects from bone metastasis in women with early breast cancer. In addition, in postmenopausal women with endocrine responsive breast cancer ZA prolongs DFS. The exact mechanism is still unclear. We have therefore investigated the effect of increasing concentrations of ZA in breast cancer cell lines in the absence or presence of estradiol to mimic the hormonal environment *in vitro*.

**Materials and methods:**

Using assays for cell proliferation (EZ4U, BrdU) and cell death (Annexin/PI), we have analyzed the dose-dependent antiproliferative and pro-apoptotic effects of ZA in two hormone sensitive cell lines (MCF-7 and T47D) and a hormone insensitive, triple negative cell line (MDA-MB-231) in the presence of 0, 1 and 10 nM estradiol.

**Results:**

In the absence of estradiol, ZA exerts dose-dependent antiproliferative and pro-apoptotic antitumor effects in both, hormone sensitive (MCF-7, T47D) and -insensitive (MDA-MB-231) breast cancer cell lines (p<0.0001). In the presence of estradiol, the antitumoral effect of ZA was significantly decreased only in the hormone sensitive MCF-7 and T47D cell lines (p = 0.0008 and p = 0.0008, respectively).

**Conclusion:**

We have demonstrated that estradiol impairs the antiproliferative and proapoptotic effect of ZA in hormone sensitive, but not in hormone insensitive breast cancer cell lines. Our findings provide a possible explanation for the differential effect of ZA on DFS in pre- and postmenopausal patients with hormone sensitive early breast cancer, which has been demonstrated clinically. We further hypothesize that endocrine insensitive tumors such as triple negative breast cancer (TNBC) should benefit from ZA irrespective of their menopausal status.

## Introduction

More than 50% of breast cancer patients present with early-stage endocrine-sensitive disease. In this subgroup, breast cancer outcomes have improved significantly over time partly because of the increasing availability of highly efficacious adjuvant therapies. Aromatase inhibitors and Tamoxifen have been shown to increase DFS in premenopausal breast cancer patients but these therapies are burdened with bone loss [1]. Favorable preclinical and clinical evidence suggesting amelioration of therapy-induced loss of bone density and prevention of skeletal events by intravenous application of bisphosphonates such as zoledronic acid in the adjuvant and metastatic settings, respectively [2–6], prompted in-depth analyses of ZA for clinical use against breast cancer.

Methodological heterogeneity has complicated the interpretation of trials examining adjuvant ZA in early breast cancer. Many of the completed trials investigated adjuvant bisphosphonate therapy for its effect on bone health [7–12]. Unfortunately, however, in most studies early treatment was compared with delayed administration rather than assessing the difference between with absolute presence and absence of bisphosphonate. In other studies, primary endpoints were used that were not related to breast cancer outcomes. The only two phase III trials that considered cancer-specific primary endpoints and compared adjuvant therapy with and without ZA (ABCSG-12 and AZURE) independently suggested that adjuvant ZA can improve breast cancer outcome specifically in patients with low levels of estrogen and after induced or natural menopause [13–15]. Meta-analyses of trials using adjuvant ZA or other bisphosphonates appear to confirm these findings [6,16–18].

Nevertheless, it has not yet been clarified which biological subtype of breast cancer will maximally benefit from ZA, nor has the relationship between efficacy of ZA to menopausal status in estrogen sensitive and–insensitive subtypes been investigated in detail.

This prompted us to conduct an *in vitro* analysis on possible differences of the antitumor effects of ZA in hormone sensitive (MCF-7 and T47D) and hormone insensitive, triple negative (MDA-MB-231) breast cancer cells in the presence or absence of estradiol.

## Materials and methods

### Breast cancer cell lines

We analyzed the antitumor effect of ZA in the presence or absence of exogenous estradiol on the hormone sensitive human breast cancer cell lines MCF-7 and T47D and on the hormone insensitive, triple negative human breast cancer cell line MDA-MB-231. All cell lines were purchased from the American Type Culture Collection (ATCC, Manassas, VA) and tested for the absence of *Mycoplasma* (Venor GeM, Minerva Biolabs, Berlin, Germany). ZA was purchased from Sigma-Aldrich (St. Louis, MO, USA).

### Cell culture

Breast cancer cell lines were seeded at a density of 2x10^6^ cells in T75 flasks and cultured in RPMI 1640 medium containing 10% heat-inactivated fetal bovine serum (FBS), 1% penicillin-streptomycin and 1% L-Glutamine to 80% confluence for use in all assays. The cells were maintained at 37°C in a humidified atmosphere with 5% CO_2_ and split weekly. Medium and supplements were purchased from Gibco^TM^ (Grand Island, NY). To achieve an estrogen-free environment cells were cultured in RPMI 1640 complete medium supplemented with 10% Charcoal/Dextran Treated Fetal Bovine Serum (HyClone^TM^, UT, USA), 1% penicillin-streptomycin and 1% L-glutamine for 5 days before treatment in all assays. Cells were harvested on day 6 and day 8 after estradiol and ZA treatment.

### Proliferation assay (EZ4U)

Proliferation assays were performed over a period of 15 days using culture medium consisting of RPMI 1640 with 10% charcoal-treated fetal calf serum (FCS-Charcoal), 1% penicillin-streptomycin and 1% L-Glutamine without and with ß-Estradiol (Sigma Aldrich, St. Louis, MO, USA) at 1 nM or 10 nM. Cell numbers were examined using a Casy cell counter (OLS, Bremen, Germany) and hemocytometer improved Neubauer (Roth, Karlsruhe, Germany) as well. After 6 and 8 days of culture a significant rise of proliferation was observed in estrogen sensitive MCF-7 and T47D, but not in estrogen insensitive MDA-MB-231 cells (data not shown).

Cell viability given as optical density was determined using a formazan dye assay (Biomedica, Vienna, Austria) as previously described [19].

### Determination of DNA synthesis by incorporation of BrdU

DNA replication was measured by BrdU. Following the manufacturers protocol, cycling cells were incubated for 48 hours with 5-bromo-2´deoxyuridine (BrdU), a synthetic analogue of thymidine, which incorporates into newly synthesized genomic DNA during the S-phase of mitosis [20].

### DNA flow cytometry

To examine the effect of ZA on apoptosis, we determined the content of cellular DNA using a fluorescent DNA-binding dye followed by flow cytometry (Abcam, Cambridge, UK). During apoptosis, genomic DNA is cleaved into smaller fragments. This is a specific marker of apoptosis and can be used to quantitate apoptosis. Using flow cytometry, PI (propidium iodide) stained cells will stain less intensely and show a peak below the G1 peak- the Sub-G1 peak. We performed the DNA flow cytometry according to the manufacturers protocol [21].

### Annexin V/PI staining

Annexin V Apoptosis Detection eFluor 450 (Affymetrix, eBioscience, San Diego, USA) was performed according to the manufacturers protocol: After dilution of 10X Binding Buffer to 1X using distilled water (1 ml 10X Binding Buffer + 9 mL dH20), we washed the cells once in PBS and once in 1X Binding Buffer. We then resuspended the cells in 1X Binding Buffer at 1x10^6^/ml and added 5 μl of fluorochrome-conjugated Annexin V to 100 μl of the cell suspension. After incubation for 15 minutes at room temperature, cells were washed in 1X Binding Buffer and resuspended in 200 μl of 1X Binding Buffer. We then added 5 μl of PI staining solution and analyzed the samples by flow cytometry [22].

### Statistical analysis

The antiproliferative and pro-apoptotic effects of ZA depending on dose, cell line and presence/absence of estradiol were determined. Analysis of Variance (ANOVA) was used to compare the effects of dose, cell line and estradiol on cell death and cell proliferation. The interaction between cell line and estradiol was assessed and reported if statistically significant. The significance level was 5%. The calculations were done in R 3.3.3.

## Results

### Estrogen sensitivity of the three cell lines

**Fig 1:**
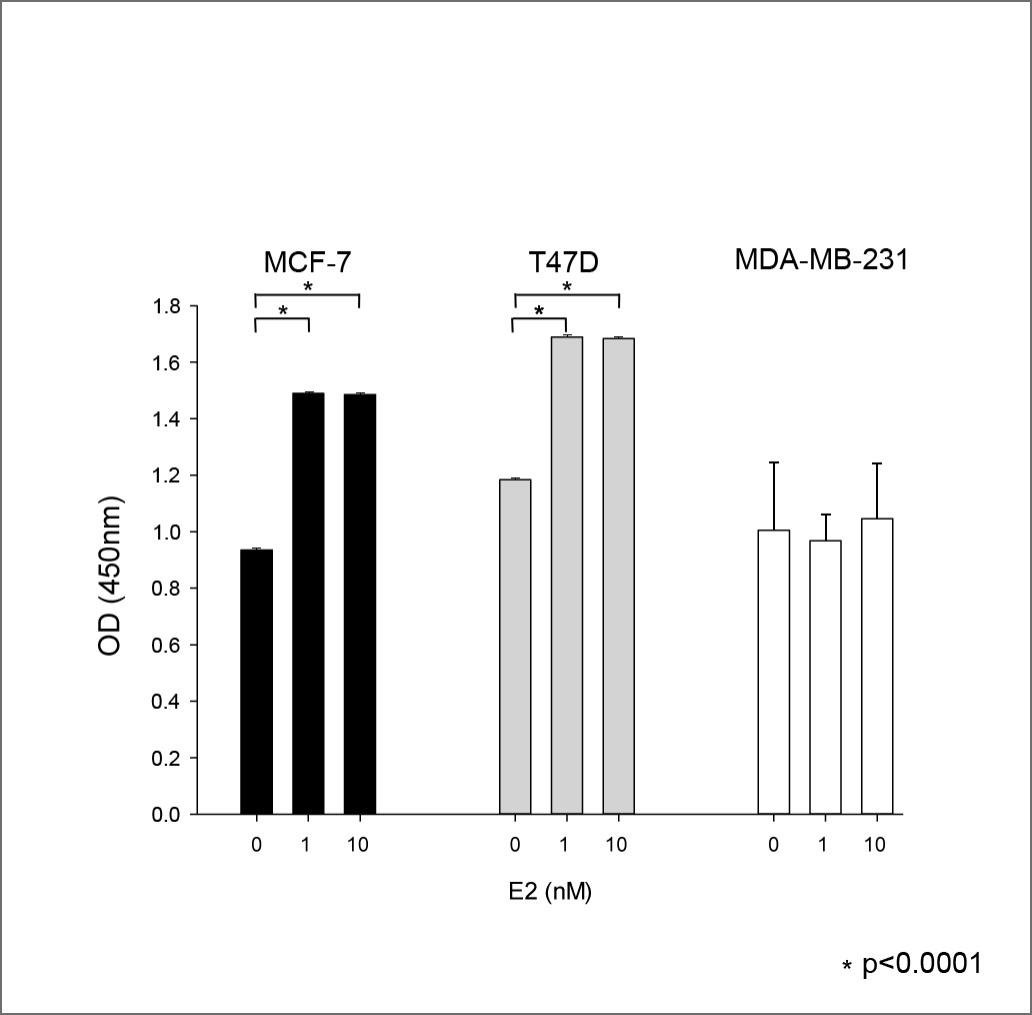
Proliferative effect of estradiol. Proliferative effect of 0, 1 and 10nM estradiol on the three cell lines MCF-7 (black), T74D (grey) and MDA-MD-231 (white) in the absence of ZA (EZ4U). Abbreviations: OD = optical density.

As expected, in the absence of ZA we found a significant proliferative stimulus by adding different doses of estradiol (0nM, 1nM, 10nM) after 5 days of estrogen depletion only in the two hormone sensitive cell lines MCF-7 and T47D (EZ4U; p<0.0001), while there was no significant increase in proliferation in the triple negative cell line MDA-MB-231. There was no significant difference in the proliferative effect between 1nM and 10nM estradiol in the hormone sensitive cell lines MCF-7 and T47D (p = n.s.) (Fig 1).

### Dose-dependent antiproliferative effect of ZA

**Fig 2:**
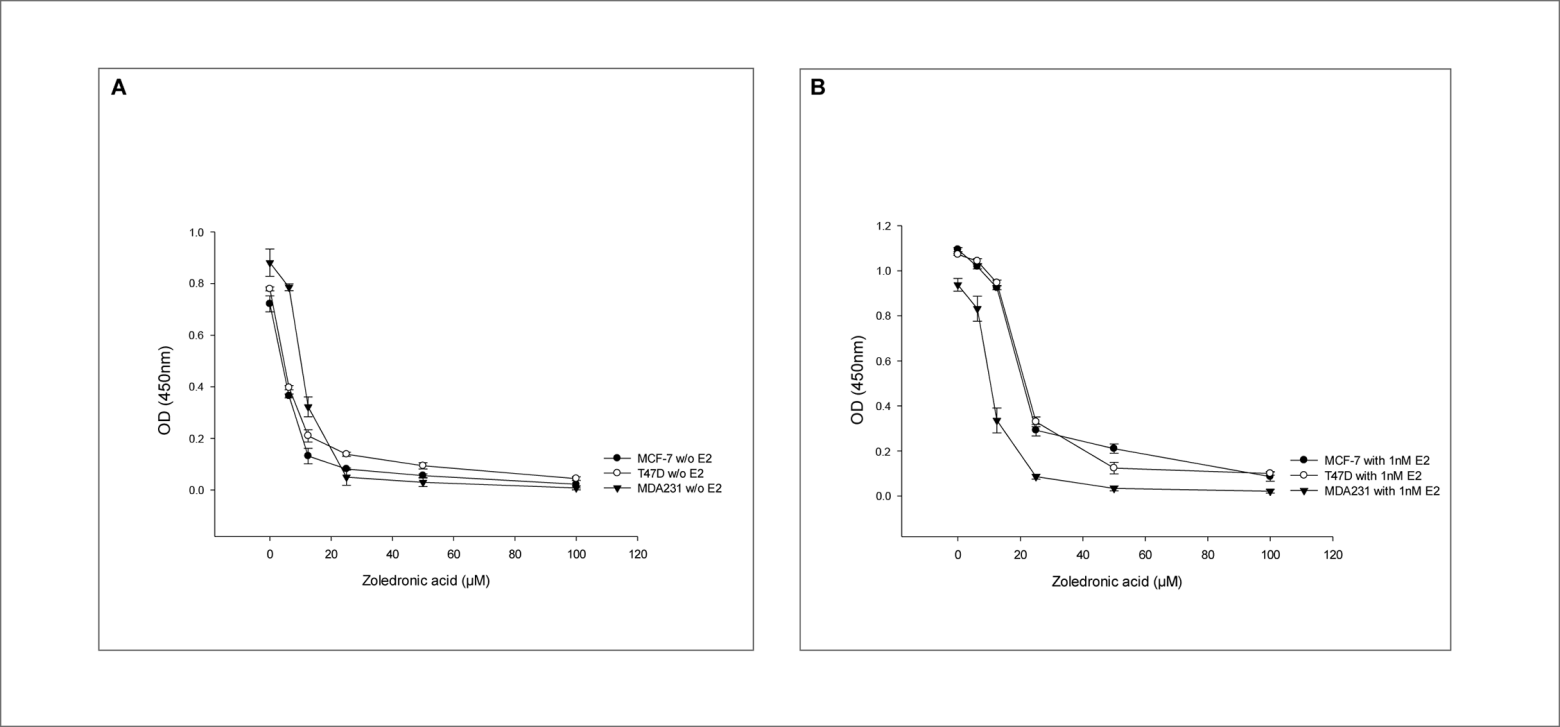
Dose-dependent antiproliferative effect of ZA. Dose-dependent antiproliferative effect of ZA on the three cell lines in the absence (A) and the presence (B) of 1nM estradiol (EZ4U). Abbreviations: OD = optical density.

Fig 2 shows the effect of increasing concentrations of ZA on the three cell lines MCF-7, T47D and MDA-MB-231 in the abscence (A) and in the presence (B) of 1nM estradiol. In the absence of estradiol, cell proliferation is suppressed in a dose-dependent manner in all three cell lines in the range between 0 and 20 μM ZA and completely inhibited by further increases (i.e. 50 and 100μM) (Fig 2A). The same dose-dependent decrease in cell proliferation is shown in the range between 0 and 20μM ZA in the presence of 1nM estradiol. However, in comparison to the hormone insensitive MDA-MB-231 cell line, at a dose of 12.5 μM ZA the antiproliferative effect is abbrogated in the hormone sensitive cell lines MCF-7 and T47D (Fig 2B).

We have therefore chosen ZA concentrations of 0, 6.25 and 12.5 μM to investigate the effect of ZA on the three cell lines in more detail.

**Fig 3:**
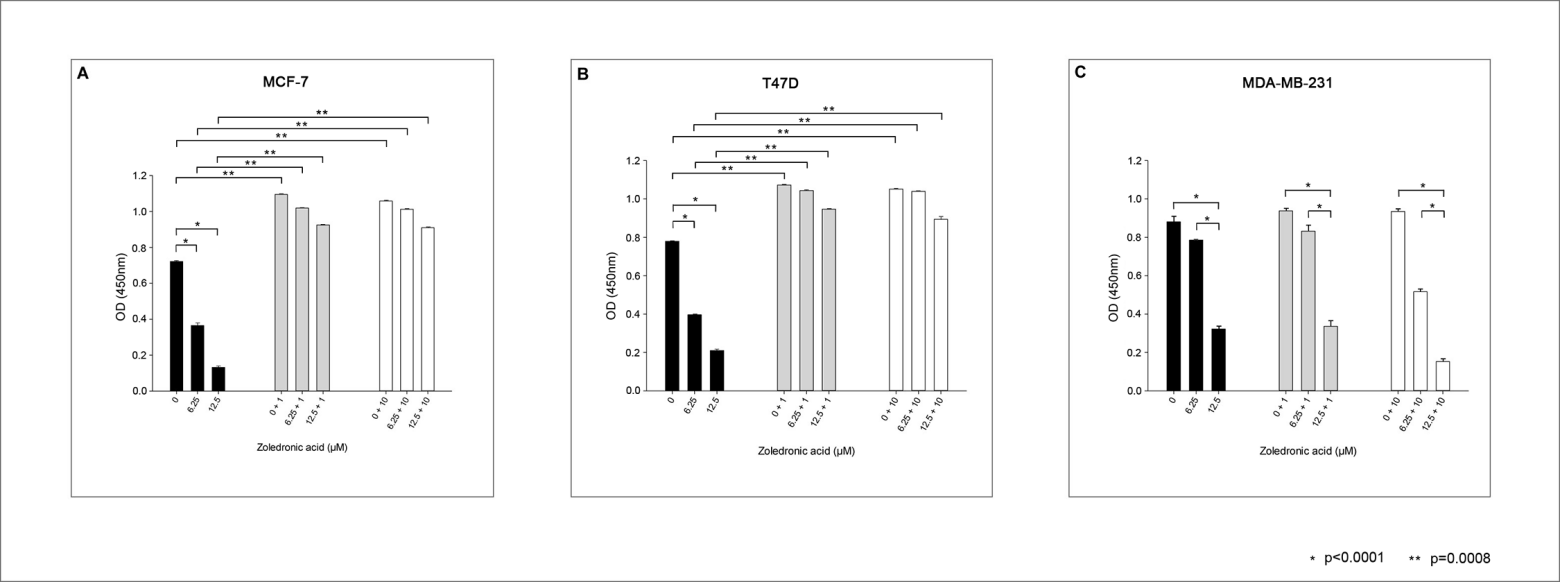
Antiproliferative effect of ZA in the absence or presence of different concentrations of estradiol. Antiproliferative effect of rising concentrations of ZA (0, 6.25 and 12.5μM) in the three cell lines MCF-7 (A), T47D (B) and MDA-MB-231 (C) in the absence (black bars) or the presence of 1nM (grey bars) and 10nM (white bars) estradiol (EZ4U).

Fig 3 shows the antiproliferative effect of ZA on the three cell lines in the absence and the presence of estradiol. In the absence of estradiol we found a significant and dose-dependent antiproliferative effect of ZA in all three cell lines (p<0.0001). In the presence of 1nM and 10nM estradiol the dose-dependent antiproliferative effect of ZA was significantly reduced in the two hormone sensitive cell lines MCF-7 and T47D (p = 0.0008), while this had no impact on the antiproliferative effect in the hormone insensitive MDA-MB-231 cell line (p = n.s.).

Remarkably, in the absence of estradiol the antiproliferative effect of increasing concentrations of ZA was stronger in hormone sensitive MCF-7 and T47D cells when compared to hormone insensitive MDA-MB-231 cells (p<0.0001). The antiproliferative effect of ZA in triple negative MDA-MB-231 cells was significantly stronger when administered at a higher dosage (p = <0.0001). (Fig 3)

### Effect of ZA on DNA synthesis

**Fig 4:**
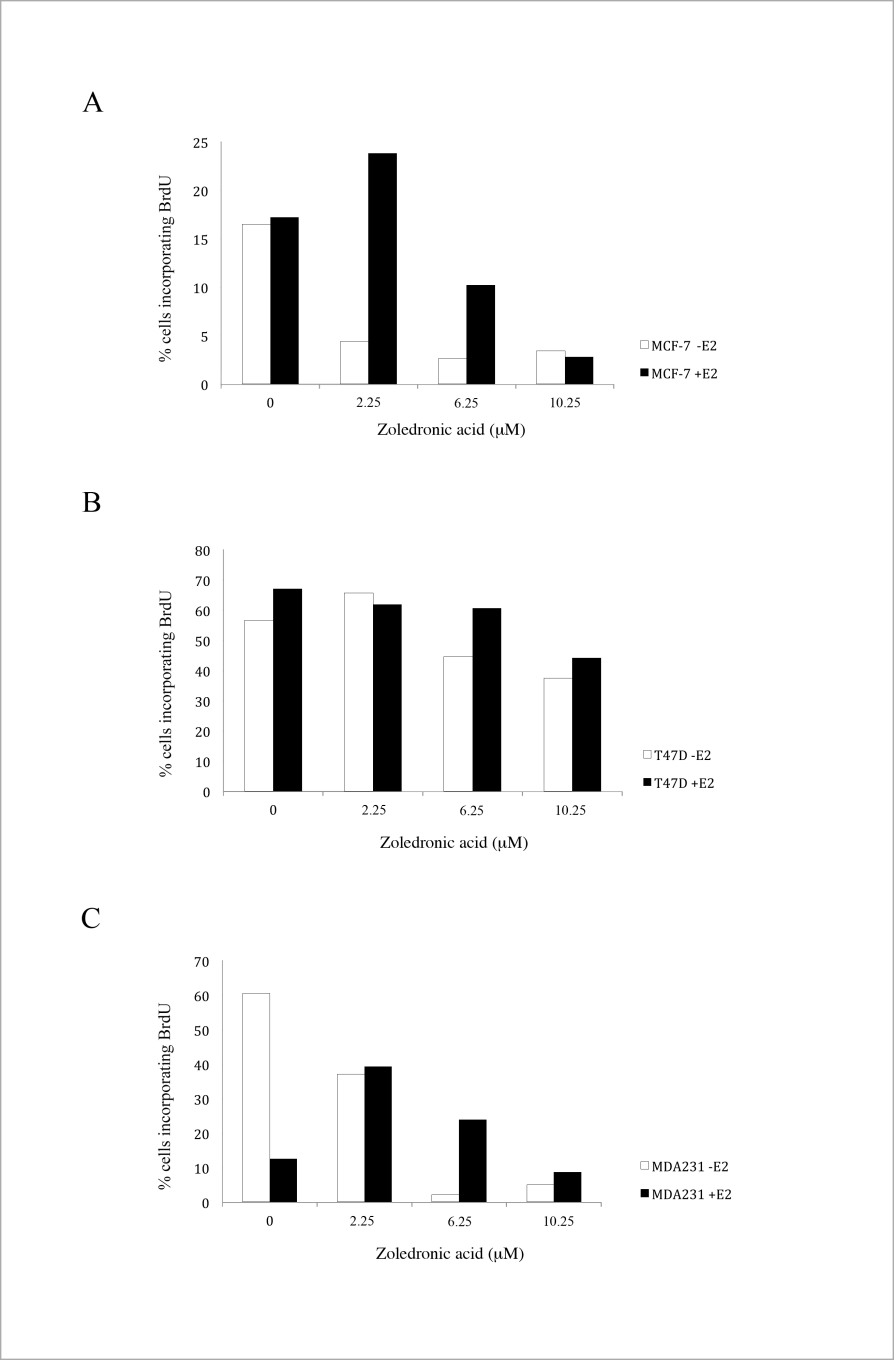
Effect of ZA on DNA synthesis. Effect of rising concentrations of ZA on DNA synthesis in the three cell lines MCF-7 (A), T74D (B) and MDA-MB-231 (C) in the absence (blue bars) and the presence (red bars) of 1nM estradiol (BrdU).

Using BrdU staining we analyzed the effect of ZA on DNA synthesis. As depicted in Fig 4, we showed a dose-dependent effect of ZA on DNA synthesis with a significant protective effect of the addition of estradiol in the two hormonsensitive cell lines MCF-7 and T-47D.

### ZA and its effect on cell cycle

**Fig 5:**
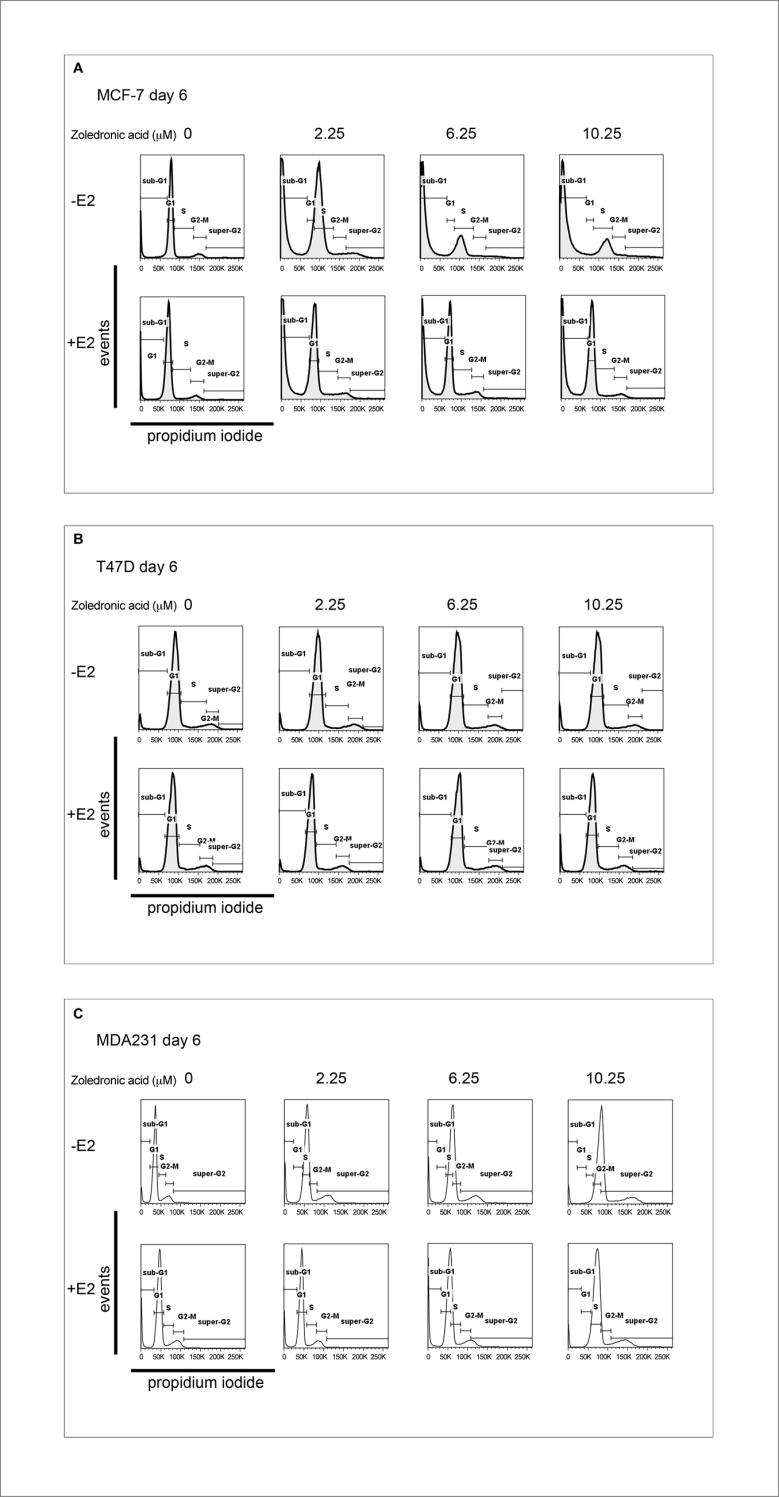
Cell cycle analysis by DNA content. Dose-dependent pro-apoptotic effect of ZA in the three cell lines MCF-7 (A), T47D (B), MDA-MB-231 (C) in the absence or the presence of 1nM estradiol shown by the Sub-G1-Peak.

We then performed a cell cycle analysis by DNA content and found a dose-dependent pro-apoptotic effect of ZA in all three cell lines shown by the sub-G1-peak in Fig 5 (p = 0.004).

This effect was stronger in the two hormone sensitive cell lines than in the triple negative cell line (p = 0.0019) without a significant influence of the addition of estradiol in all three cell lines (p = 0.104).

### Apoptotic effect of ZA

**Fig 6:**
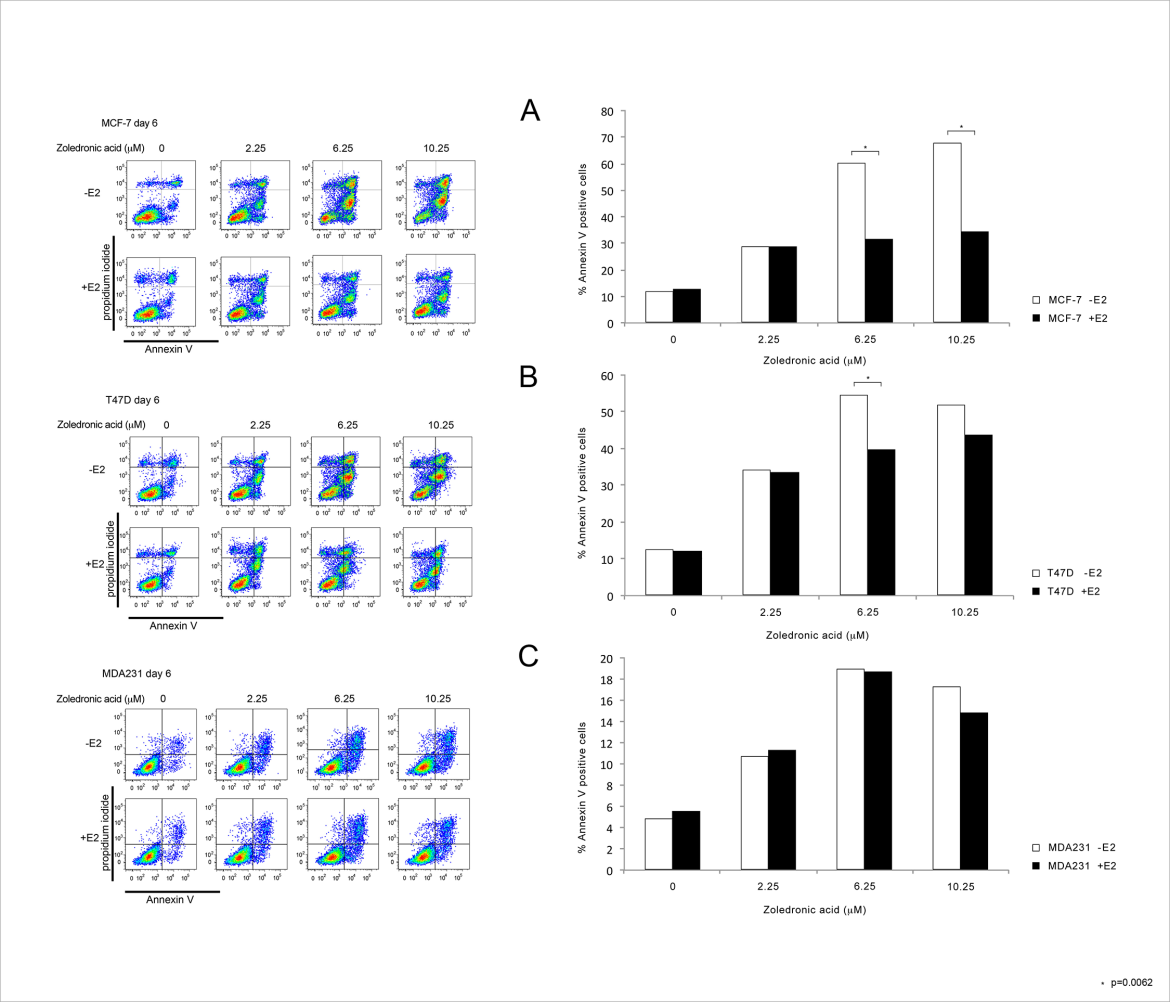
Pro-apoptotic effect of ZA measured by Annexin/PI staining. Pro-apoptotic effect of rising concentrations of ZA in the three cell lines MCF-7 (A), T47D (B), MDA-MB-231 (C) in the absence or the presence of 1nM estradiol measured by AnnexinV/PI staining.

To investigate if the pro-apoptotic effect of ZA is dependent on dosage, the addition of estradiol and the tumor subtype, we performed an Annexin V apoptosis detection test as shown in Fig 6. We found a significant dose dependent pro-apoptotic effect of ZA in all three cell lines (p = 0.0001). The addition of estradiol lead to a significant decrease in the amount of apoptotic cells only in the two hormone sensitive cell lines (p = 0.0062).

## Discussion

Bisphosphonates improve bone mineral density (BMD) in osteoporotic women and have also been shown to ameliorate anticancer treatment-induced decrease of BMD and bone fracture [23–25]. Importantly, apart from lowering therapy-related toxicity bisphosphonates have also been shown to exert direct antineoplastic effects on tumor cells [26–36]. However, in the adjuvant setting it is still not clear whether all patients or only those with hormone receptor positive disease after menopause would benefit from bisphosphonates.

The Early Breast Cancer Trialists´ Collaborative Group (EBCTCG) performed a meta-analysis of 26 clinical trials including over 18,000 women with early breast cancer randomly assigned to receive bisphosphonates versus no treatment or placebo. The reduction in recurrence, distant recurrence, and breast cancer mortality were of borderline statistical significance, although a reduction in bone recurrence was found (10-year risk 7.8 versus 9.0 percent, first-event rate ratio 0.83; 95% CI 0.73–0.94). A subset analysis of the EBCTCG meta-analysis revealed that use of bisphosphonates was associated with a statistically significant reduction in the risk of recurrence, bone recurrence, and breast cancer mortality in women with non-functioning ovaries (either because they were postmenopausal, or premenopausal but with subsequent ovarian suppression or ovariectomy). Women with functioning ovaries had no apparent effect on any outcome [37].

Moreover, there is some preclinical evidence demonstrating that reproductive hormones can reduce the efficacy of bisphosphonates against cancer cells disseminated to the bone. The effects of ZA on the growth of disseminated MDA-MB-231 breast cancer cells in bone were compared in ovariectomised mice or sham-operated mice. ZA decreased the number of detectable tumors in bone only in the ovariectomised animals [38].

Experimental work addressing the possible influence of tumor biology on the direct anticancer efficacy of ZA is still lacking. Most previous studies evaluated the effects of ZA in hormone sensitive breast cancer patients [26,36,37]. In contrast, no data are available demonstrating whether hormone insensitive breast cancer patients would benefit from ZA in their neoadjuvant or adjuvant regimens.

Preclinical evidence presented by Verdijk et al demonstrated that sensitivity of ZA is most pronounced in estrogen receptor negative MDA-MB-231 cells [39]. Similarly, in the EBCTCG meta-analysis a tendency for an even stronger benefit of ZA in estrogen receptor negative breast cancer patients was observed. Albeit, this trend was statistically not significant, which is probably due to the fact that in the bisphosphonate arm the population of ER negative patients was limited to 107/1964 patients, whereas in the control arm the proportion of ER negative cancers was markedly higher (135/1684) [37]. In a case-control analysis with 3,731 postmenopausal breast cancer patients, Rennert G et al found a similar advantageous, but statistically underpowered, effect of oral bisphosphonates on overall survival in patients with ER positive as well as ER negative and Her-2 positive disease [40].

In a meta-analysis of randomized trials looking at the effects of neoadjuvant chemotherapy with or without ZA on pathological response, significant higher pathologic complete response rates (pCR) were observed only in the subgroup of postmenopausal patients, whereas only a trend towards higher pCR rates was shown in triple negative breast cancer patients [41].

Here we have demonstrated that ZA exerts direct antitumoral effects in breast cancer cells, irrespective of hormone receptor status and hormone-sensitivity or -resistance. ZA reduced cell multiplication and DNA synthesis, and caused cell cycle arrest and programmed cell death in all cell lines tested. Obviously, these ZA-mediated antitumor responses were not modulated by the presence or absence of exogenous estradiol in the triple negative hormone resistant MDA-MB-231 cells, whereas in hormone receptor positive MCF 7 and T47-D cells, administration of estradiol significantly reduced the antiproliferative and pro-apoptotic effects of ZA. In general, ZA was found to cause accumulation of the cells in the S-phase of the cell cycle, which is consistent with the findings of Neville-Webbe HL et al [42].

Taken together, herein we present first experimental evidence demonstrating that ZA exerts direct dose-dependent anti-proliferative and pro-apoptotic effects in both estrogen sensitive and–insensitive breast cancer cells *in vitro*. Evidently, a deteriorating effect of exogenous estradiol on the anticancer efficacy of ZA was detected only in hormone sensitive, but not in hormone insensitive breast cancer cells. Consequently, our findings underscore previous clinical evidence showing that postmenopausal patients with hormone sensitive breast cancer benefit most from adjuvant ZA, whereas patients with hormone insensitive breast cancer may benefit from adjuvant ZA independent of menopausal status. Based on these data specific clinical studies in various patient subgroups characterized by tumor biology and menopausal status are warranted in order to identify those patients that in fact would or would not benefit from (neo-) adjuvant ZA.

## Supporting information

S1 Fig Influence of tamoxifen on cell proliferation. Cell proliferation of the three cell lines MCF-7, T47D, MDA-MB231 with and without estradiol and tamoxifen.
(PDF)

S1 Table EZ4U raw data. (XLSX)

S2 Table BrdU raw data. (XLSX)

S3 Table Annexin V/PI staining raw data. (XLSX)
