# Supplementary figures and images for: Estradiol impairs the antiproliferative and proapoptotic effect of Zoledronic acid in hormone sensitive breast cancer cells in vitro
Source: PLoS One. 2017 Sep 25;12(9):e0185566. doi: 10.1371/journal.pone.0185566 (PMC5612728; doi:10.1371/journal.pone.0185566)

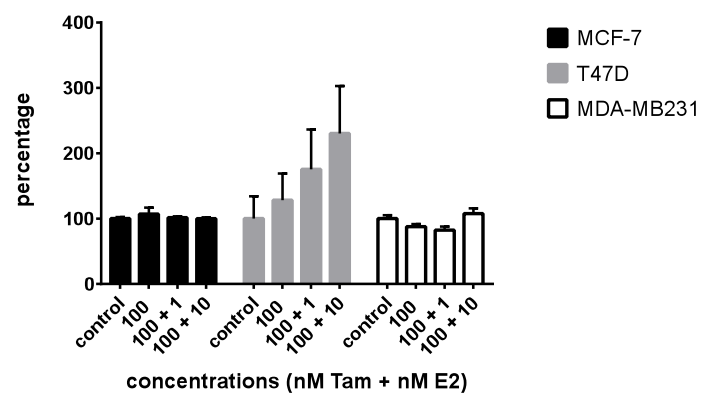

Supplement: S1 Fig — Cell proliferation of the three cell lines MCF-7, T47D, MDA-MB231 with and without estradiol and tamoxifen. (PDF) [file pone.0185566.s001.pdf]
